# Complementarity of surgical therapy, photobiomodulation, A-PRF and L-PRF for management of medication-related osteonecrosis of the jaw (MRONJ): an animal study

**DOI:** 10.1186/s12903-022-02275-2

**Published:** 2022-06-18

**Authors:** Mohammad Reza Jamalpour, Shiva Shahabi, Mehdi Baghestani, Abbas Shokri, Shokoofeh Jamshidi, Salman Khazaei

**Affiliations:** 1 Department of Oral and Maxillofacial Surgery, Dental Implants Research Center, Hamadan University of Medical Sciences, Hamadan, Iran; 2 Dental Implants Research Center, Hamadan University of Medical Sciences, Hamadan, Iran; 3 Department of Oral and Maxillofacial Surgery, Hamadan University of Medical Sciences, Hamadan, Iran; 4 Department of Oral and Maxillofacial Radiology, Dental Implants Research Center, Hamadan University of Medical Sciences, Hamadan, Iran; 5 Department of Oral and Maxillofacial Pathology, Dental Implants Research Center, Hamadan University of Medical Sciences, Hamadan, Iran; 6 Department of Epidemiology, Research Center for Health Sciences, Hamadan University of Medical Sciences, Hamadan, Iran

**Keywords:** Photobiomodulation therapy, Laser therapy, Platelet-rich fibrin, Medication-related osteonecrosis of the jaw, Zoledronate

## Abstract

**Background:**

This study aimed to evaluate the complementarity of surgical therapy, photobiomodulation (PBM), advanced platelet-rich fibrin (A-PRF), and Leukocyte and platelet-rich fibrin (L-PRF) for the management of medication-related osteonecrosis of the jaw (MRONJ).

**Methods:**

Sixty rats underwent injection of zoledronate followed by left mandibular first and second molar extractions to induce MRONJ lesions. All rats were examined for the signs of MRONJ 8 weeks post-dental extraction. Forty-nine rats with positive signs of MRONJ were appointed to seven different groups as follows: control (Ctrl); surgery alone (Surg); surgery and PBM (Surg + PBM); surgery and A-PRF insertion (Surg + APRF); surgery and L-PRF insertion (Surg + LPRF); surgery, A-PRF insertion, and PBM (Surg + APRF + PBM); surgery, L-PRF insertion, and PBM (Surg + LPRF + PBM). Euthanasia was carried out 30 days after the last treatment session. The lesions' healing was evaluated clinically, histologically, and radiographically. Data were analyzed using STATA software version 14, and the statistical significance level was set at 5% for all cases.

**Results:**

According to the present study, A-PRF and L-PRF treatment resulted in significant improvements in clinical, histological, and radiographical parameters compared to the Ctrl group (*P* < 0.05). The PBM also decreased wound dimensions and the number of empty lacunae compared to the Ctrl group (*P* < 0.05). Surg + APRF + PBM and Surg + LPRF + PBM were the only groups that presented a significantly higher mean number of osteocytes (*P* < 0.05). No significant differences were observed between A-PRF and L-PRF treatment groups (*P* > 0.05).

**Conclusions:**

Surgical resection followed by applying A-PRF or L-PRF reinforced by PBM showed optimal wound healing and bone regeneration in MRONJ lesions.

## Introduction

Medication-related osteonecrosis of the jaw (MRONJ) is a side effect of antiresorptive and antiangiogenic drugs widely prescribed for metabolic and oncologic diseases of the skeletal system, such as osteoporosis, Paget's disease, multiple myeloma, and bone metastases of the malignant neoplasms [1, 2]. While these medications may significantly affect the diagnosed diseases' treatment, they can induce necrotic lesions of the jawbones, particularly following invasive dental treatments such as tooth extraction [3].

It is believed that the combined effect of extreme bone remodeling suppression, bacterial infection, angiogenesis inhibition, and immunological dysfunction may lead to oral mucosa's incomplete healing and jaw osteonecrosis [4, 5]. The jaw osteonecrosis association with bisphosphonates usage was first presented by Marx [6] in 2003. The American Association of Oral and Maxillofacial Surgeons (AAOMS) has defined MRONJ by the simultaneous presence of three following characteristics: persistently exposed bone or a bone that can be probed via an intraoral or extraoral fistula in the maxillofacial region for more than 8 weeks; pervious or current antiangiogenic or antiresorptive treatment; and no history of radiotherapy or jaws' metastatic diseases [7]. MRONJ may affect the patients' quality of life and overall health by causing significant pain, infection, and functional disabilities.

The current MRONJ treatment goals are controlling the pain, secondary infection, and necrotic areas progression while supporting ongoing medication treatments. Surgical and non-surgical conservative therapies are the two main conventional methods for MRONJ treatment [7, 8]. The gold-standard treatment for MRONJ has remained a controversial subject. Due to ineffective healing and high recurrence rate, recent studies have focused on upgrading conventional methods by adding adjunctive therapies such as photobiomodulation (PBM), hyperbaric oxygen, ozone, teriparatide, and autologous platelet concentrations (APC) [9–11].

Bio-stimulation through laser irradiation appears to be a simple and non-invasive way to relieve pain and improve wound healing [12, 13]. It has been discovered that photobiomodulation ameliorates bone formation by increasing the osteoblasts' proliferation and differentiation, calcium deposition, collagen type 1 formation, adenosine triphosphate (ATP) synthesis, and growth factors secretion [14].

Leukocyte and platelet-rich fibrin (L-PRF) and advanced platelet-rich fibrin (A-PRF) are second generations of APCs obtained by eliminating additives in centrifugation [15]. They are three-dimensional fibrin scaffolds capable of releasing numerous cytokines and growth factors and hence great stimulators for local tissue regeneration [16, 17]. The release of numerous growth factors is reported to be significantly higher in A-PRF than in L-PRF [18]. The reason behind this is the decreased centrifugal force during the preparation of A-PRF (1500 rpm, 14 min) compared to L-PRF (2700 rpm, 12 min), which shifts fewer cells to the bottom of the tube, leading to higher numbers of leukocytes in the platelet-rich fibrin (PRF) layer [19]. We used both preparation methods to determine if these extra released growth factors affect the healing process of the MRONJ lesion.

Given the challenges of MRONJ management, we aimed to evaluate the effect of using adjunctive therapies, including PBM, A-PRF, and L-PRF, on the MRONJ healing and new bone formation in the affected areas.

## Materials and methods

### Animals

This animal study was approved by the Ethics Committee of Hamadan University of Medical Science (IR.UMSHA.REC.1399.448) and conducted in accordance with relevant guidelines and regulations. All methods are reported in accordance with ARRIVE guidelines [20].

60 male Wistar Albino rats (300–350 g) were provided and adapted to the laboratory temperature and humidity for 10 days before the study commenced. The animals were provided with a standard diet of rat pellets and water ad libitum.

### MRONJ induction

Zandi et al. [21] protocol for MRONJ induction with an 83% success rate was applied in this study. The animals received an intraperitoneal injection of 0.06 mg/kg zoledronate (Zolena, Ronak Pharmaceutical, Saveh, Iran) once a week for 6 weeks, followed by the left mandibular first and second molar extractions at the end of week 6. The extractions were conducted under general anesthesia with an intraperitoneal injection of 75 mg/kg ketamine hydrochloride (Ketamine Hydrochloride, Laboratoires Sterop, Brussels, Belgium) and 7.5 mg/kg midazolam (Midazolam, Chemidarou Industrial Company, Tehran, Iran). Once the sedation was verified, rats were placed supine, and the teeth were luxated and extracted using dental surgical forceps. For analgesia, 2 mg/kg ketorolac (Ketorolac, Caspian Tamin Pharmaceutical, Gilan, Iran) was subcutaneously injected post-extraction. For three postoperative days, animals were fed crushed pellets as a soft diet, and amoxicillin drops (50 mg/ml, Amoxicillin, Tehran Chemie, Tehran, Iran) were added to their drinking water (1.5 mg/ml of water). The zoledronate injections were continued for another 6 weeks after the teeth extractions.

### Study groups

Eight weeks post-extraction, all rats were examined for signs of MRONJ defined by AAOMS [7]. During intraoral examinations, mesiodistal (MD) and buccolingual (BL) dimensions of the wound and exposed bone area were measured with a graduated probe. The difference between the wound and the exposed bone area dimensions was that the wound dimensions also entailed the inflamed mucosa surrounding the exposed bone area, which had distinguishable color and consistency compared to the normal mucosa. Moreover, the presence of extraoral and intraoral fistulas was noted. Eventually, 49 rats with positive signs of MRONJ were randomly appointed to seven study groups (Table 1).

**Table 1:** Study groups (n = 7, each)

Groups	Intervention
Ctrl	No intervention (control)
Surg	Surgical resection alone
Surg + PBM	Surgical resection + PBM
Surg + APRF	Surgical resection + A-PRF
Surg + LPRF	Surgical resection + L-PRF
Surg + APRF + PBM	Surgical resection + A-PRF + PBM
Surg + LPRF + PBM	Surgical resection + L-PRF + PBM

### Surgical resection

All treatment groups, except the control group, underwent surgical resection. General anesthesia was induced by an intraperitoneal injection of 75 mg/kg ketamine hydrochloride (Ketamine Hydrochloride, Laboratoires Sterop, Brussels, Belgium) and 7.5 mg/kg midazolam (Midazolam, Chemidarou Industrial Company, Tehran, Iran) [21]. A mucoperiosteal flap was elevated and mobilized to expose the necrotic bone areas and enable further free-tension closure. After excision of inflamed wound margins, the necrotic bone was removed with a surgical round bur. The surgical extent was limited by intraoperative parameters such as reaching bleeding bone margins and eliminating any sharp edge. In Surg + APRF, Surg + LPRF, Surg + APRF + PBM, and Surg + LPRF + PBM groups, the surgical defect was first covered with the PRF membrane. Subsequently, tension-free primary wound closure was obtained using two simple interrupted 4-0 nylon (Nylon, Supa, Tehran, Iran) sutures. These sutures helped immobilize the membrane, and the obtained covering mucosa prevented the membrane from being washed by the saliva. The defects were repaired through primary closure with 4-0 nylon (Nylon, Supa, Tehran, Iran) sutures in other groups. The sutures were removed 10 days after the surgery. Postoperative care was done as previously described for teeth extractions.

### Photobiomodulation

In Surg + PBM, Surg + APRF + PBM, and Surg + LPRF + PBM, photobiomodulation was performed using an 808 nm diode laser (3L-IR, Hamerz, Tehran, Iran) on 1st, 3rd, 5th, 7th, and 10th postoperative days, according to the parameters mentioned in Table 2 [12].

**Table 2:** Photobiomodulation parameters

Parameter	Value/characteristics
Manufacturer	Hamerz, Tehran, Iran
Model identifier	3L-IR
Year produced	2019
Number and type of emitters	One Diode laser
Wavelength	808 nm
Pulse mode	Continuous wave (CW)
Power	500 mW
Beam spot size at the target	0.28 cm^2^
Irradiance at target	1785.71 mW/cm^2^
Exposure duration	120 s
Radiant exposure	5 J/cm^2^
Radiant energy	1.4 J
Number of points irradiated	One
Area irradiated	0.28 cm^2^
Application technique	Noncontact mode 0.5–1 cm distance from the target
Number and frequency of treatment sessions	Five sessions on postoperative 1st, 3rd, 5th, 7th, and 10th days
Total radiant energy over the entire treatment course	7 J

### Preparation of A-PRF and L-PRF

In Surg + APRF, Surg + LPRF, Surg + APRF + PBM, and Surg + LPRF + PBM groups, following the surgical resection, 2 ml of blood was collected from the retro-orbital sinus into the specified sterile tubes using a capillary pipette (plain glass-based vacuum tube for A-PRF and glass-coated plastic tube for L-PRF) [22]. Instant centrifugation was done at 1500 rpm for 14 min and at 2700 rpm for 12 min to prepare A-PRF and L-PRF, respectively. After centrifugation, the tube content was removed, and the PRF layer was separated from the top acellular plasma layer and the bottom red blood cell layer [15, 19]. Then, the PRF layer was compressed to form a membrane ready to be inserted into the defect [23]. The size of the PRF membrane was decided intraoperatively based on the surgical defect extension.

### Clinical examinations

**Fig. 1:**
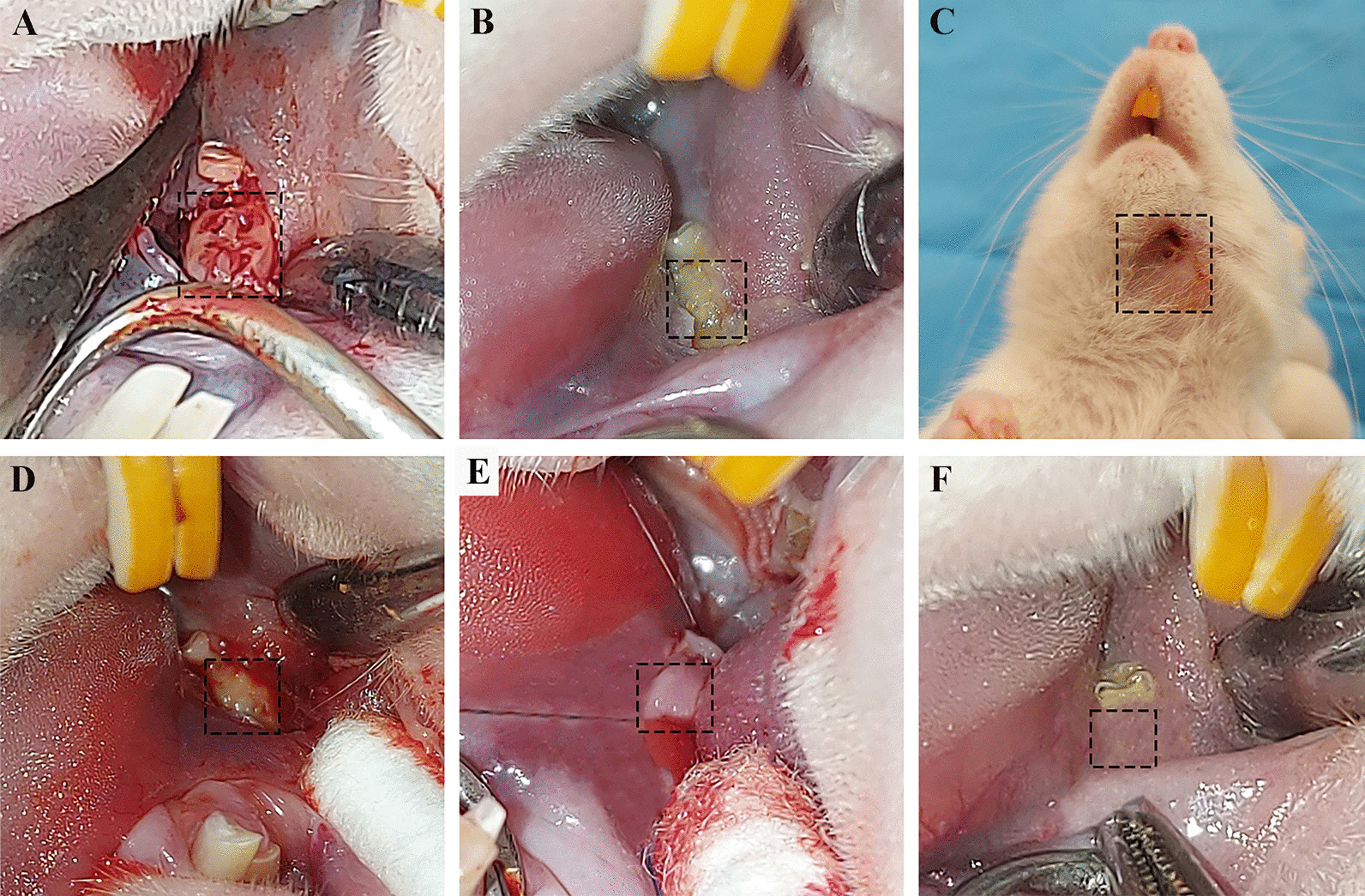
Clinical findings. **A** Empty sockets of the extracted teeth, **B** induced MRONJ lesion, **C** extraoral fistula, **D** healthy bleeding bone margins after surgical resection, **E** inserted PRF membrane before primary wound closure, and **F** healed MRONJ lesion

Euthanasia was carried out 30 days after the last PBM therapy session. The presence of extraoral and intraoral fistulas, MD and BL wounds dimensions, and MD and BL dimensions of possible bone exposure areas were recorded. Two blinded observers graded the mucosal healing as follows: unsatisfactory (presence of exposed bone), satisfactory (mucosal coverage with distinguishable color and consistency from the healthy mucosa), and highly satisfactory (healthy mucosal coverage) (Fig. 1).

### Radiographical evaluation

Following mandibulectomies and additional tissue elimination, radiographic images of the left side hemimandibles were taken. The hemimandibles were placed parallel to the radiographic digital film, the experimental sites were marked with opaque pointers, and digital periapical radiographs were obtained with a MinRay (Soredex, Tuusula, Finland) operating at 60 kVp, 6 mA, and 0.12 s exposure time. In each obtained radiographical image, a 20 × 20 pixels square was selected from the center of the marked site, with the upper edge starting 5 pixels below the markers. The cropped squares were inputted to MATLAB software v7.11 (MathWorks, Cambridge, MA, USA). For each image, the gray value of every pixel was determined as a number between zero and 255. The value zero signified the lowest and 255, the highest reduction of X-ray beams. Finally, the mean and standard deviation of all pixels' gray values were calculated. Image bone density analyses were conducted using the mean gray values of the selected squares from the center of the experimental sites.

### Histological examination

Hemimandibles were fixed in 10% formalin solution for 72 h and later decalcified with Ethylenediaminetetraacetic acid (EDTA) for 30 days. After demineralization, all samples were embedded in paraffin, sectioned buccolingually at the experimental site (5 µm thick, four sections per sample), and stained with hematoxylin and eosin. Two blinded pathologists performed the histological examinations. The average number of newly formed osteocytes and empty lacunae per 25 mm^2^ was calculated in five different fields, utilizing a 10 × 10 mm eyepiece gride reticle (light microscopy, 400 × magnification). Descriptive evaluations of the epithelial tissue integrity, vascularization, and inflammatory infiltration were done under 400 × magnification in five different fields. The vascularization was presented by a four-point scale (0: absent, 1: slight, 2: moderate, and 3: marked), as well as inflammatory infiltration (0: absent, 1: 1 to 100 cells as slight, 2: 100 to 250 cells as moderate, and 3: more than 250 cells as severe). The epithelial tissue integrity was graded as unsatisfactory (no epithelial tissue formation), satisfactory (interrupted epithelial tissue formation), and highly satisfactory (complete epithelial tissue coverage).

### Statistical analysis

Data were expressed as absolute frequency, percentage, and mean ± standard deviation and were analyzed using STATA 14 (StataCorp LP., Lakeway, Texas, USA). The interobserver agreement was analyzed using the kappa coefficient (κ-values > 0.75: excellent, κ-values < 0.40: low, and 0.40 < κ-values < 0.75: moderate agreement). Pearson's chi-square test or Fisher's exact test (when the expected values in any contingency table cells were less than five) determined the association between qualitative variables. The quantitative variables were compared using the one-way ANOVA test, and multiple comparisons were analyzed using Tukey’s post hoc test. The statistical significance level was set at 5%.

## Results

Overall, the animals tolerated the experimental period fairly. Four rats died before the teeth extractions due to excessive weight loss caused by the zoledronate administration. Other animals remained in good health, achieved proper hemostasis, and recovered well from anesthesia.

### Clinical findings

Pre-treatment examinations revealed no statistically significant difference in the mean MD and BL wound dimensions and the mean MD and BL exposed bone area dimensions among study groups (*P* > 0.05). Therefore, the Ctrl group data was considered representative of pre-treatment data to be compared with the post-treatment results of each study group. Additionally, there was no statistically significant difference in the fistulas number among study groups (one intraoral and one extraoral fistula in each group, *P* > 0.05).

**Fig. 2:**
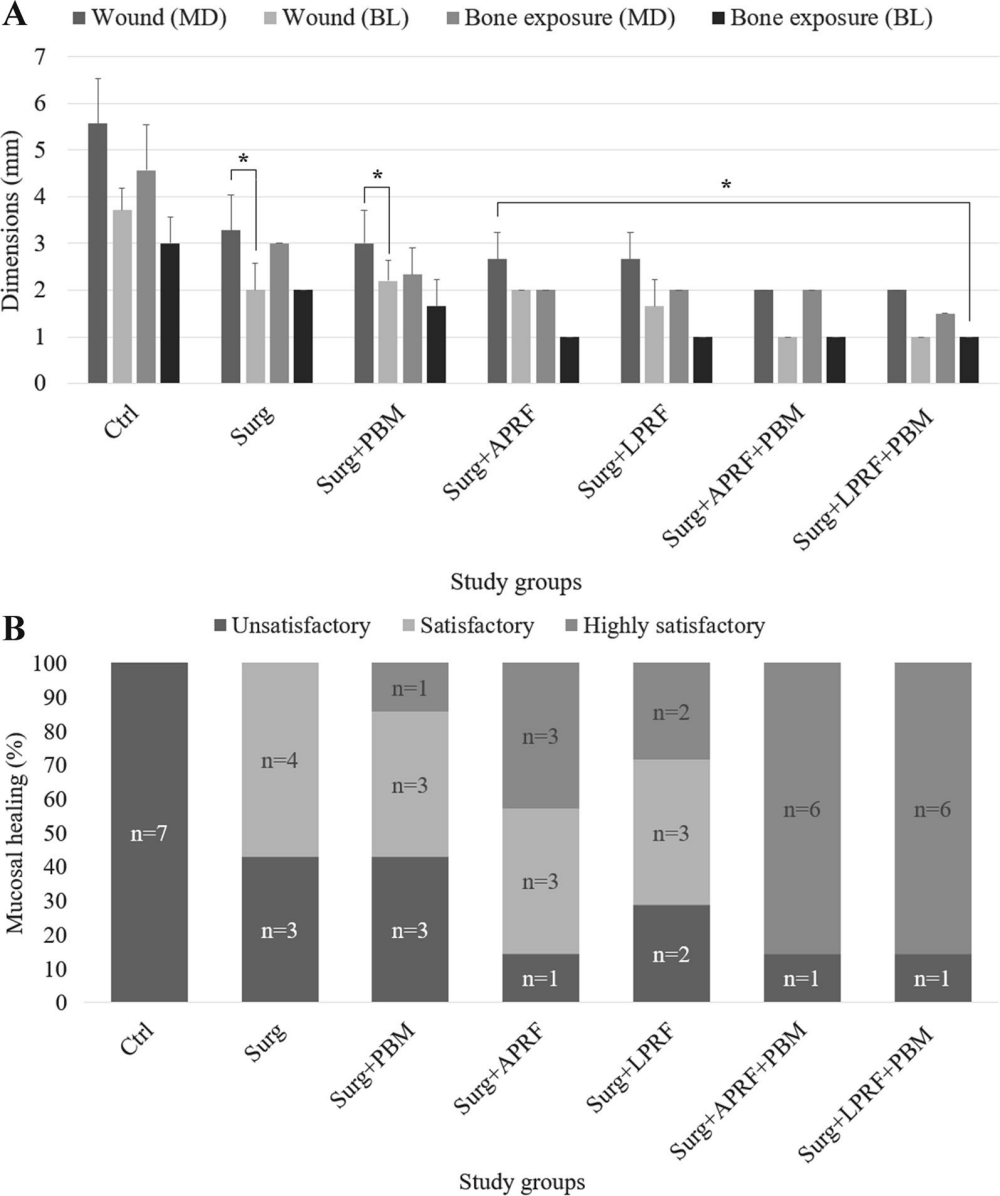
Post-treatment clinical results. **A** Mesiodistal (MD) and buccolingual (BL) dimensions of the wounds and the bone exposure areas in different study groups (*, statistically significant difference in relation to the Ctrl group), and **B** mucosal healing in study groups

Interobserver agreement in clinical examinations’ assessment was excellent (κ = 0.93, *P* < 0.001). Post-treatment clinical examinations showed that the mean MD and BL bone exposure area dimensions in Surg + APRF, Surg + LPRF, Surg + APRF + PBM, and Surg + LPRF + PBM were significantly lower than in the Ctrl (*P* < 0.05). Also, the mean MD and BL wound dimensions were significantly lower in all treatment groups than in the Ctrl (*P* < 0.05). The intraoral fistula was healed in Surg + APRF, Surg + LPRF, Surg + APRF + PBM, and Surg + LPRF + PBM, and the extraoral fistula was healed in Surg + APRF, Surg + APRF + PBM, and Surg + LPRF + PBM. The optimal mucosal healing outcome was observed in Surg + APRF + PBM and Surg + LPRF + PBM. Six out of seven cases of these groups showed highly satisfactory mucosal healing. Satisfactory mucosal healing was the most frequent observation in the Surg group (n = 4). All cases of the Ctrl group (n = 7) showed unsatisfactory mucosal healing (Fig. 2).

### Radiographical findings

**Fig. 3:**
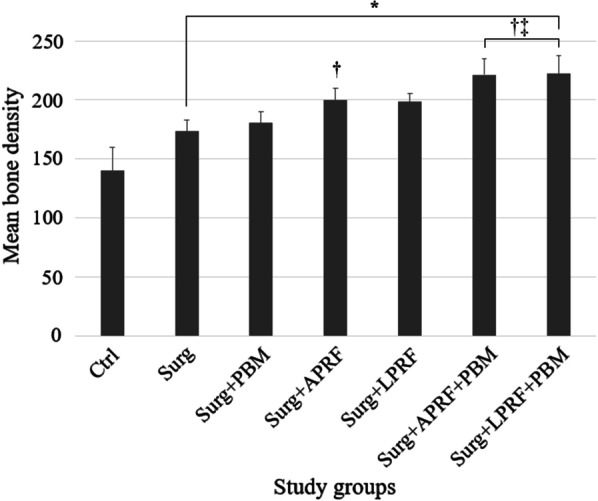
Mean bone density of the experimental sites in different study groups (*, statistically significant difference in relation to the Ctrl; †, statistically significant difference in relation to the Surg; ‡, statistically significant difference in relation to the Surg + PBM)

All treatment groups had higher mean bone density than the Ctrl. The mean bone densities of Surg + APRF (199.65 ± 9.8), Surg + APRF + PBM (221.3 ± 13.45), and Surg + LPRF + PBM (222.32 ± 14.94) were significantly higher than the Surg (173.31 ± 9.75) (*P* < 0.05). Surg + APRF + PBM and Surg + LPRF + PBM were the only groups that showed significantly higher mean bone density in comparison to the Surg + PBM (180.61 ± 9.37) (*P* < 0.05) (Fig. 3, Table 3).

**Table 3:** Comparative table of bone density between study groups

Study groups	Bone density	
	Mean dif	*P*-value^*^
*Ctrl*		
Surg	33.30	0.003
Surg + PBM	40.60	< 0.001
Surg + APRF	59.88	< 0.001
Surg + LPRF	58.64	< 0.001
Surg + APRF + PBM	81.29	< 0.001
Surg + LPRF + PBM	82.31	< 0.001
*Surg*		
Surg + PBM	7.30	0.977
Surg + APRF	26.57	0.034
Surg + LPRF	25.34	0.051
Surg + APRF + PBM	47.98	< 0.001
Surg + LPRF + PBM	49	< 0.001
*Surg* + *PBM*		
Surg + APRF	19.27	0.261
Surg + LPRF	18.03	0.339
Surg + APRF + PBM	40.68	< 0.001
Surg + LPRF + PBM	41.7	< 0.001
*Surg* + *APRF*		
Surg + LPRF	− 1.23	1
Surg + APRF + PBM	21.40	0.157
Surg + LPRF + PBM	22.43	0.12
*Surg* + *LPRF*		
Surg + APRF + PBM	22.64	0.113
Surg + LPRF + PBM	23.66	0.084
*Surg* + *APRF* + *PBM*		
Surg + LPRF + PBM	1.02	1

^*^Tukey's post hoc test

### Histological findings

**Fig. 4:**
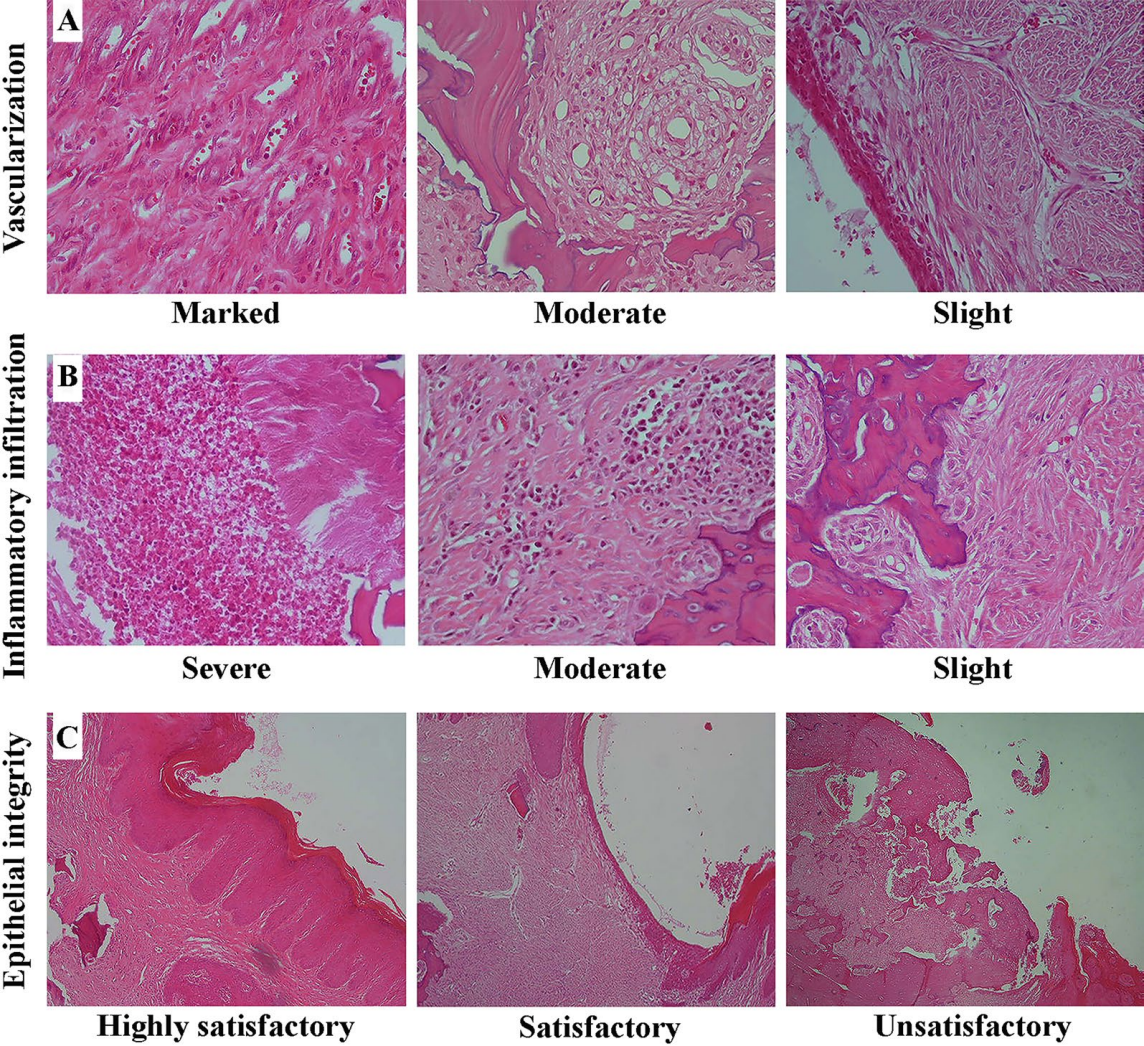
Histological images. **A** From left to right: marked, moderate, and slight vascularization (400 × magnification), **B** from left to right: severe, moderate, and slight inflammatory infiltration (400 × magnification), and **C** from left to right: highly satisfactory, satisfactory, and unsatisfactory epithelial tissue integrity (100 × magnification)

For histological findings, the Interobserver agreement was excellent (κ = 0.88, *P* < 0.001). There were significant differences in vascularization and inflammatory infiltration among groups (*P* < 0.001). All specimens (n = 7) from Surg + PBM, Surg + APRF, Surg + LPRF, Surg + APRF + PBM, and Surg + LPRF + PBM had slight vascularization. 5 cases showed moderate vascularization in the Surg, and 2 cases showed slight vascularization. The Ctrl group cases' vascularization was ranked as moderate (n = 5) and marked (n = 2) (Fig. 4A).

Severe inflammatory infiltration was seen in specimens from Ctrl (n = 7), Surg (n = 5), and Surg + PBM (n = 2). Moderate inflammatory infiltration was the main observation in Surg + PBM (n = 5), Surg + APRF (n = 7), Surg + LPRF (n = 7), and Surg + APRF + PBM (n = 5). Slight inflammatory infiltration was only detected in Surg + APRF + PBM (n = 2) and Surg + LPRF + PBM (n = 5) (Fig. 4B).

A statistically significant difference in epithelial tissue integrity was found between the study groups (*P* = 0.002). The highly satisfactory epithelial integrity was observed only in Surg + APRF + PBM (n = 2) and Surg + LPRF + PBM (n = 3). Unsatisfactory epithelial integrity was dominant in Ctrl (n = 7), Surg (n = 6), and Surg + PBM (n = 5). There were fewer cases of unsatisfactory epithelial integrity in Surg + APRF (n = 3), Surg + LPRF (n = 2), Surg + APRF + PBM (n = 1), and Surg + LPRF + PBM (n = 1). Notably, satisfactory epithelial integrity was mostly reported in Surg + APRF (n = 4), Surg + LPRF (n = 5), Surg + APRF + PBM (n = 4), and Surg + LPRF + PBM (n = 3), which were treated by PRF (Fig. 4C).

Furthermore, study groups exhibited significant differences in the mean number of osteocytes and empty lacunae (*P* < 0.001). The mean empty lacunae numbers were significantly lower in PRF-treated groups, including Surg + APRF (14.42 ± 4.23), Surg + LPRF (11.57 ± 1.9), Surg + APRF + PBM (6.14 ± 4.3), and Surg + LPRF + PBM (8.28 ± 5.61) than in Surg (24.28 ± 4.23) and Ctrl (31 ± 3.36). Additionally, the mean empty lacunae numbers in the Surg + APRF + PBM and Surg + LPRF + PBM were significantly lower than in Surg + PBM (20.28 ± 7.67). The Surg + PBM group's mean empty lacunae number was significantly lower than the Ctrl group (31 ± 3.36) (*P* < 0.05).

**Fig. 5:**
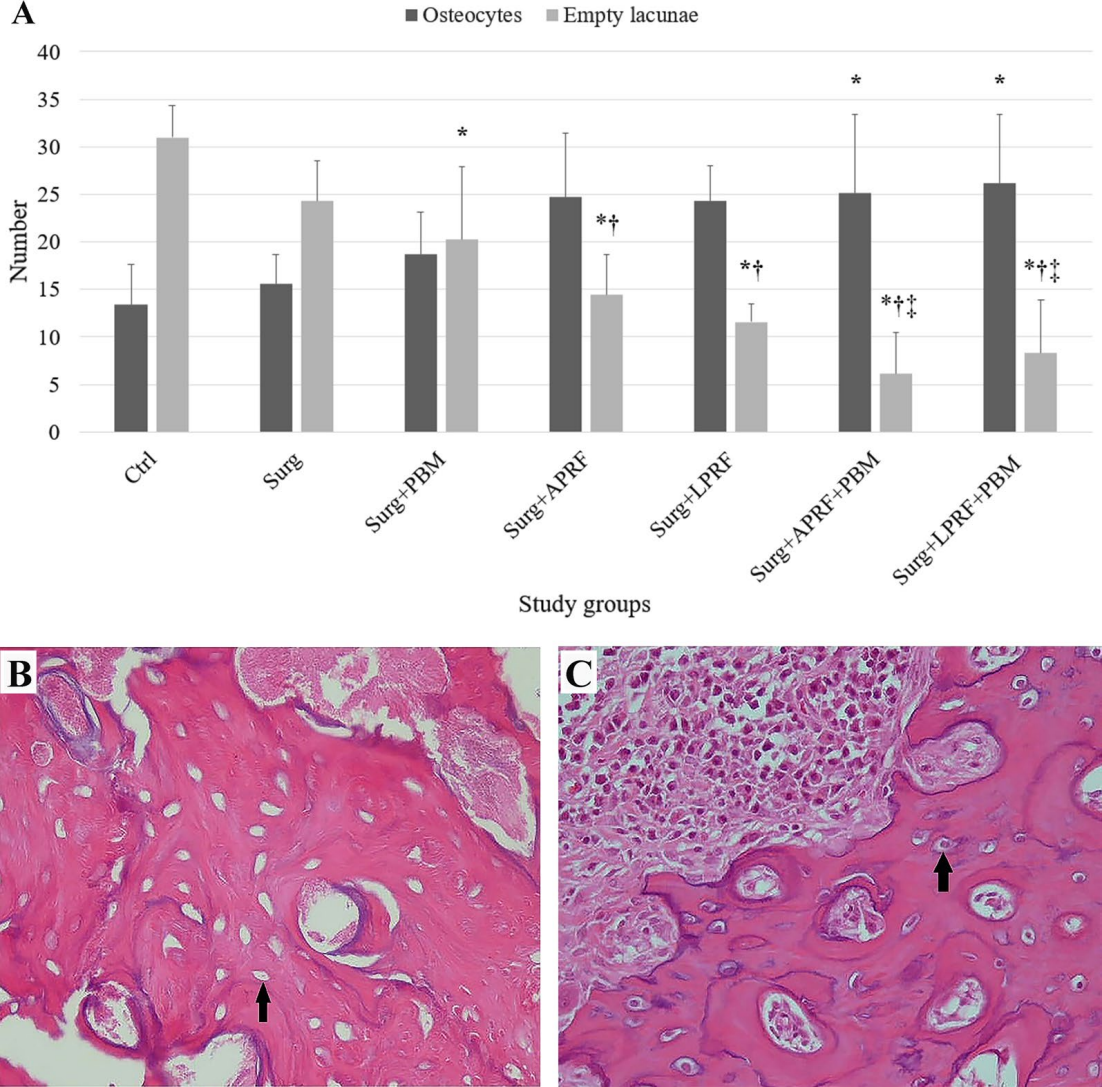
**A** The mean number of osteocytes and empty lacunae in the experimental sites in different study groups (*, statistically significant difference in relation to the Ctrl; †, statistically significant difference in relation to the Surg; ‡, statistically significant difference in relation to the Surg + PBM), **B** histological image of non-vital bone tissue containing empty lacunae marked by the black arrow (magnification: 400 ×), and **C** histological image of vital bone tissue characterized by lacunae filled with osteocytes marked by the black arrow (magnification: 400 ×)

The only significant difference in the mean osteocytes number was observed in Surg + APRF + PBM and Surg + LPRF + PBM, which presented a higher mean osteocytes number (25.14 ± 8.25 and 26.14 ± 7.22, respectively) than the Ctrl (13.42 ± 4.23) (*P* < 0.05) (Fig. 5, Table 4).

**Table 4:** Comparative table of osteocytes number and empty lacunae number between study groups

Study groups	Osteocytes number		Empty lacunae number	
	Mean dif	*P*-value^*^	Mean dif	*P*-value*
*Ctrl*				
Surg	2.14	0.998	− 6.71	0.351
Surg + PBM	5.28	0.805	− 10.71	0.018
Surg + APRF	11.28	0.054	− 16.57	< 0.001
Surg + LPRF	10.85	0.072	− 19.42	< 0.001
Surg + APRF + PBM	11.71	0.040	− 24.85	< 0.001
Surg + LPRF + PBM	12.71	0.019	− 22.71	< 0.001
*Surg*				
Surg + PBM	3.14	0.982	− 4	0.869
Surg + APRF	9.14	0.203	− 9.85	0.038
Surg + LPRF	8.71	0.254	− 12.71	0.002
Surg + APRF + PBM	9.57	0.160	− 18.41	< 0.001
Surg + LPRF + PBM	10.57	0.087	− 16	< 0.001
*Surg* + *PBM*				
Surg + APRF	6	0.694	− 5.85	0.521
Surg + LPRF	5.57	0.763	− 8.71	0.097
Surg + APRF + PBM	6.42	0.621	− 14.14	0.001
Surg + LPRF + PBM	7.42	0.446	− 12	0.005
*Surg* + *APRF*				
Surg + LPRF	− 0.42	1	− 2.85	0.972
Surg + APRF + PBM	0.42	1	− 8.28	0.133
Surg + LPRF + PBM	1.42	1	− 6.14	0.462
*Surg* + *LPRF*				
Surg + APRF + PBM	0.85	1	− 5.42	0.612
Surg + LPRF + PBM	1.85	0.999	− 3.28	0.945
*Surg* + *APRF* + *PBM*				
Surg + LPRF + PBM	1	1	2.14	0.994

*Tukey’s post hoc test

## Discussion

To improve the MRONJ healing process, we need to reduce the medications' inhibitory effects by accelerating bone and soft tissue regeneration with adjuvant therapies. There are various reports of adjuvant therapies; however, these treatments' efficacy in comparison or combination with one another has yet to be established [9, 10]. The success rate of such treatments depends on their required frequency, costs, complexity, and patient collaboration. Hence, we selected two available and cost-effective adjuvant therapies (PBM and PRF therapy) to investigate.

The necrotic bone's presence causes constant soft tissue irritation and thus interferes with proper healing [24]. Exposed bone and epithelialization absence result in persistent and recurrent infections postponing the healing [25]. Therefore, necrotic bone elimination is essential. According to Hayashida et al. [26], the first treatment choice should be surgical therapy, and prolonged conservative therapy may decrease the patient's quality of life and exacerbate the lesion. Accordingly, we applied surgical resection to all experimental groups as the primary treatment.

Based on our findings, surgical treatment (Surg group) resulted in lower mean wound dimensions and higher bone density than the Ctrl. However, there were no substantial improvements in other clinical and histological parameters. Most specimens from the Surg group had severe inflammation, moderate vascularization, and unsatisfactory epithelial integrity. Consequently, surgical resection is required to provide the underlying healthy margins for tissue regeneration, but it cannot enhance the regeneration alone.

Adjuvant PBM therapy resulted in similar outcomes as the surgery. Although, PBM therapy resulted in lower mean empty lacunae than the Ctrl group. The inflammation and vascularization of most Surg + PBM group specimens were also reduced, which could be justified by the granulation tissue maturation. Since there was no obvious bone formation progression, we supposed that the fibrotic tissue formation replaced the granulation tissue.

Similar to our study, Vescovi et al. [27] suggested that PBM applications stimulate angiogenesis and soft tissue healing. Based on the systematic review by de Souza Tolentino et al. [28], from 246 cases who underwent laser therapy, 64.2% showed improved symptoms, and 39.8% were healed completely. Many studies have confirmed the positive PBM effects on tissue regeneration, including promoting cell proliferation, calcium deposition, and angiogenesis [14]. In this study, we could not detect PBM stimulation effects on bone regeneration which might be explained by the suppression effect of zoledronate on bone remodeling [29]. Likewise, Ervolino et al. [30] observed that PBM therapy could not alter the bone remodeling in extraction sites of rats treated with zoledronate.

Notably, PBM outcomes are highly dependent on various factors, such as laser wavelength, laser settings, and sessions' frequency and duration. The pre-treatment MRONJ stage also affects the PBM therapy's success rate.

The present study also investigated the PRF therapy effect on MRONJ healing. Based on our findings, in the cases treated with PRF (Surg + APRF and Surg + LPRF), the mean wound and bone exposure area dimensions were considerably lower than the Ctrl group. All extraoral and intraoral fistulas were healed except for one case. Most cases showed satisfactory and highly satisfactory mucosal healing. Enhanced bone remodeling was detected in these groups, which was expected due to inflammation reduction, vascularization reduction, and granulation tissue maturation. The epithelial integrity was also better than the previous groups.

PRFs affect MRONJ remission by mechanical and inflammatory protections and enduring bio-activator properties [31]. Their fibrin architecture provides a scaffold that stores cells such as platelets and prevents the direct toxicity of bone-released bisphosphonates on the soft tissue by acting as a barrier between bone and oral mucosa [32, 33]. Trapped platelets in these fibrins are responsible for releasing growth factors, upregulating osteoprotegerin and alkaline phosphatase, and osteoblasts' proliferation [34, 35].

Unlike PBM therapy, PRFs' regenerative properties persist for a significant time throughout the healing process (usually 7 to 28 days) and do not need repetition [15]. This might explain the better outcomes observed in a 1-month follow-up after PRF treatment rather than PBM.

In many case reports and a few clinical trials on MRONJ, the PRFs' application has shown promising results [32, 36, 37]. Based on Kim J.-W. et al.'s study [38], 26 out of 34 patients showed complete MRONJ resolution after L-PRF treatment. Giudice et al. [39] investigated the PRF's efficacy after surgery compared to surgery alone at three different time points. Their results exhibited significant differences in mucosal integrity, infection absence, and pain resolution between treatment groups in favor of PRF at the 1-month follow-up.

PRFs' preparation is an economical and straightforward process with no technical difficulties. They are chemical-free products from patients' blood that can be easily handled. Considering the mentioned advantages, PRF therapy is an appropriate adjuvant treatment for MRONJ.

We also investigated the efficacy of simultaneous PRF and PBM therapies (Surg + APRF + PBM and Surg + LPRF + PBM). These were the only groups that showed a higher statistically significant number of osteocytes (*P* < 0.05). Moreover, we observed substantially higher mean bone density and fewer empty lacunae than Ctrl, Surg, and Surg + PBM groups. These groups showed complete healing of fistulas and 6 out of 7 cases of highly satisfactory mucosal healing. They were the only groups showing slight inflammation and highly satisfactory epithelial integrity.

These results indicate the synergic effect of PBM and PRF co-application. The PBM bio-stimulatory effects might activate PRFs' platelets, leading to enhanced growth factors releasing and tissue remodeling.

Merigo et al. [40] treated 21 MRONJ patients using platelet-rich plasma (PRP) and 808 nm laser after removing necrotic tissues by piezosurgery and Er:YAG laser. 92.85% of patients reached complete healing at 6-months follow-up. Hence, they suggested consecutive different high-technology strategies during the MRONJ treatment. Using different methods to eliminate the necrotic tissues and PRP instead of PRF hinders comparing the results between our study and theirs. However, they also supported the application of more than one adjuvant therapy.

Finally, it is worth mentioning that despite applying two different protocols to prepare PRFs (A-PRF and L-PRF), we detected no significant differences between them (*P* > 0.05).

The main limitation of our study was that due to the needed animal sacrifices for radiographical and histological evaluations, we had to use small animals (rats) with the minimal possible sample size that did not jeopardize the study statistically. Additionally, we used plain radiographs as they are more commonly available, but we recommend applying more exact radiological evaluation methods such as micro-computed tomography (micro-CT) or X-ray fluorescence.

Within the treatment selections investigated in this study, we concluded that the combination of PBM and PRF placement might be the most practical choice of MRONJ treatment. These adjuvant therapies improved clinical, histological, and radiological parameters examined in this study. PRF therapy alone revealed better outcomes than PBM alone, and we observed no substantial differences between A-PRF and L-PRF.

## Data Availability

The datasets used and/or analyzed during the current study are available from the corresponding author on reasonable request.

## Abbreviations

MRONJ: Medication-related osteonecrosis of the jaw
AAOMS: American Association of Oral and Maxillofacial Surgeons
PBM: Photobiomodulation
APC: Autologous platelet concentrations
PRF: Platelet-rich fibrin
A-PRF: Advanced platelet-rich fibrin
L-PRF: Leukocyte and platelet-rich fibrin
MD: Mesiodistal
BL: Buccolingual
EDTA: Ethylenediaminetetraacetic acid
micro-CT: Micro-computed tomography
